# Professors Brera and Coindet, &c. on Iodine

**Published:** 1823-03-01

**Authors:** 


1. *Saggio Clinico sull'Iodio, e sulle differenti sue Combinazioni e Preparazioni Farmaceutiche, &c.* i. e. *Clinical Essay on Iodine, and its different Combinations and Pharmaceutical Preparations; from Results obtained in the Clinical School of Padua, in* 1820-1821. By PROFESSOR BRERA. Octavo, pp. 106. Padua, 1822.

2. *Observations on the remarkable Effects of Iodine in Bronchocele and Scrophula: being a Translation of three Memoirs published by* J. R. COINDET, M. D. of Geneva. Octavo, pp. 32. London, 1821. Translated by J. R. JOHNSON, M. D.

BEING thoroughly convinced that it is not to the discovery of new remedies, but to the improved application of those already known, through the increase of pathological knowledge, that we are to look for any important advancement of therapeutics, we are accustomed to hear the frequent announcement of fresh accessions to the materia medica, with perfect tranquillity; and if we occasionally avail ourselves of such in our practice, it is rather in compliance with fashion than from a conviction of their superiority, much less of their exclusive power to fulfil any important indication in the treatment of diseases. Indeed, we are well convinced, that modern medicine has derived infinitely more precious benefits from many physiological and pathological discoveries, of little notoriety and less pretensions—contained probably in some few unostentatious paragraphs, than from all the multifarious additions, rejections, and revivals, which have characterized our pharmacology during the last fifty years. Still it is neither liberal nor philosophical to reject unheard the claims of these new candidates for a place in the storehouse of health; nor is it just to declare that all those which have been heard and tried are either useless or unnecessary. The great majority of such novelties unquestionably are so; yet it is but fair to admit that some few of these (for example, colchicum, croton, cubebs, prussic acid,) are at least deserving a place among the list of substances possessed of analogous powers. Each and all of these, it is true, we expect to live long enough to see consigned, like the other articles of present fashion, to a temporary oblivion, until resuscitated to gratify the unquenchable appetite of novelty, with the bag-wigs, red heels, hoops, or trunk-hose of our ancestors; still we feel that it is our duty, as ministers of health, and still more as medical annalysts, to make ourselves, and also our readers, acquainted with them as they respectively present themselves before us; and to hear and judge their claims and pretensions to our regard, be they great or small. It is on this principle that we have, in former numbers of our Journal, devoted some portion of our pages to the medicines abovementioned; and in the present article we intend to introduce to our readers another new agent, that comes before us with as high pretensions, and probably with powers as great, as any of its recent precursors. This new medicine is IODINE, which has now been somewhat more than two years known to the profession, but which has hitherto obtained from us only a very brief and imperfect notice.—(Vol. II. p. 322.)

The simple substance, Iodine, was discovered in the soda derived from the incineration of certain marine vegetables, by Mons. Courtois, in the year 1813. Several of its properties were first investigated by M. Clement; but it is principally to Sir H. Davy and Gay-Lussac that we are indebted for our knowledge of the chemical habitudes of Iodine. For the history of this discovery, and the subsequent development of the various relations of this substance we must refer to the 88th, 89th, 92d, and 93d vols. of the Annales de Chimie, and to the various scientific journals published in this country since the period of its discovery.

The first experiments on the living body with this new agent, were made by Majendie, who was led to conclude that it was not poisonous; but subsequent experiments of Orfila on animals completely establish its great virulence, in certain doses, and justified its classification among the corrosive poisons. It is to Dr. Coindet of Geneva, however, that we are entirely indebted for the introduction of iodine into medicine. This gentleman reflecting on the benefits derived from burnt sponge, from time immemorial, in the cure of bronchocele,* and on the more recent discovery of similar virtues in the preparations of the common sea-weed (fucus vesiculosus;) and on the fact that iodine is common to these and other marine productions; was led by analogy to suspect that it was to *it* that the influence of these substances in curing bronchocele was to be attributed. As he lived in the midst of a

* We are informed by Professor Brera that there is preserved in the library of St. Mark, in Venice, a copy of the works of Van Helmont, full of marginal annotations in the hand writing of our famous countryman Locke, among which is the following formula recommended for the cure of goitre:—

R. Spungiæ marinæ in carbonem combustæ uncias tres ossium sæpiæ ustorum, piperis longi, zinziberis, pyretri, gallarum, salis gemmæ, calcis testorum avorum—*ana unciam unam.* Terantur omnia simul in pulverem fin. cujus uncia semis. liquat. deglutitur paulatim *decrescente luna.*

population very subject to this complaint, he had an imme-diate opportunity of putting his conjecture to the test of experiment, and was speedily gratified by obtaining the most decisive proofs of the efficacy of the new remedy. The result of his first trials were published by Dr. Coindet in July 1820, in a memoir printed in the Bibliotheque Universelle; and this was afterwards followed by two others. These memoirs were translated into English by Dr. J. R. Johnson, and published in the form of a small pamphlet, in the winter of 1821. In his first memoir Dr. C. informs us that under the use of iodine a vast number of cases of bronchocele have been cured in a space of from six to ten weeks, and in such a way as to leave no trace of their existence; but that some of the tumours that appear goitrous resist the action of this remedy under every form of prescription; and that others are only dissipated partially, but so as to leave neither inconvenience nor deformity. He states iodine to be a most active emenagogue; and concludes by expressing his conviction "of its becoming, under skilful hands, one of the most powerful remedies with which modern chemistry has enriched the materia medica." In his second memoir, the author protests against the unjust clamour that appears to have been raised against the new remedy by the public in Geneva on the score of its dangerous effects on the living system; attributing such ill effects as may have been witnessed by others, to the incautious, indiscriminate, and popular use of it, and repeating his former opinions, strengthened by additional experience, of its great efficacy and (when properly administered) perfect safety. He here states that the neutral salt formed by the hydriodic acid and potass to which some pure iodine has been added *(ioduretted hydriodate of potass)* is the preparation most easy to manage, and is the one almost exclusively used by him. In this memoir the author enters somewhat more fully upon the effect of iodine on the animal economy, and gives some account of the symptoms developed by its continued use, or the *saturation* of the body with it, in which respect (as we shall see hereafter) it resembles mercury and arsenic. He repeats his belief that the new remedy will be found useful in amenorrhœa and other chronic diseases of the uterus; and states his having found it very successful in the cure of indolent scrophulous tumours of the glands in the neck. In his third memoir Dr. Coindet gives an account of his trials with the iodine rubbed in *externally* with lard, and states his having treated twenty-two cases of goitre in this manner, and cured more than half of these in from four to six weeks. He was led to employ the remedy externally in hopes that it would not be found to occasion the disagreeable effects occasionally resulting from its internal use, and which he seems to think owing to its local action on the mucous membrane of the stomach and bowels; and he considers that experience justified his hopes, as he says that this method "presents a sure and easy mode of employing this powerful remedy, exempt from those objections made to its internal exhibition." In this memoir he states his further experience of the iodine in enlarged scrophulous glands, and that his scccess has exceeded his most sanguine expectations. He suggests the probable benefit of a combination of this medicine with mercury in syphilitic complaints complicated with scrophula; and of the simple remedy in ovarian affections, from the analogy some of these bear to the disease of the thyroid gland.

Since the publication of Dr. Coindet's memoirs, iodine has been employed by many practitioners of eminence, as for instance, by Dr. Decarro of Vienna, Formey of Berlin, Majendie and Gimelle of Paris, Sacco and Omodei of Milan, Fenolio of Turin, and lastly, by the distinguished author of the Clinical Essay whose title is placed at the head of this article. In the hands of all of these it has been most successful in the cure of bronchocele, and has been considered by some of them as a valuable medicine in other diseases. In this country, we believe it has not had a very ample trial. We mentioned in a former number of this Journal that it had been used in one case by Dr. Kennedy of Glasgow, without success; but we learn from some communications in the Medical and Physical Journal for August and October 1822, that it has been used with great success in the case of goitrous affections common in the elevated parts of Sussex and Surry. In one instance a comparative trial of the sponge lozenges and tincture of iodine was made by a very judicious and well informed practitioner, Mr. Austin of Haslemere, and the result was in favour of the superior efficacy of the latter. In the part of the country just mentioned (Haslemere) we have the means of knowing that bronchocele prevails to a surprizing extent, affecting almost every female (and scarcely any males) among the labouring classes. In these cases the burnt sponge lozenges (prepared by Shepherd of Fleet Street) are considered by the resident medical gentlemen as of almost specific efficacy, if persevered in for a sufficient length of time, and are not found to be productive of any ill effects.

Mr. Austin, we are informed, is now engaged in giving a full trial to the iodine; and we have every reason to expect from him a judicious administration of the remedy, and a faithful history of its effects.

It was with the knowledge of the results obtained from the employment of this remedy in France, Switzerland, and Germany, that Brera determined to give it a full trial in the Clinical School of Padua in the year 1821; and in the small volume before us he has submitted to the profession the fruits of his experience. Although the author entitles this a *Clinical* Essay, and commences with a detail of cases, yet he takes occasion, in the course of his memoir, to give a more connected and comprehensive view of the general medical relations of iodine than is to be found in any other work; and as we think these details will prove much more interesting than the cases, we shall reverse the order of his Essay, and give an account of these general matters first. In doing this, as our object will be to submit to our readers an epitome of what is known respecting the new remedy, we shall neither restrict ourselves to the order nor to the substance of our author's treatise, but cull our materials from whatever other sources are accessible to us.

I. *Chemical History of Iodine.* For a complete account of this we must refer to the sources formerly mentioned, and to the work before us. We shall only here observe that iodine is a simple substance, of characters analogous to those of oxygen, chlorine, &c. solid at the ordinary temperature of the atmosphere, but volatile at a moderate degree of heat, under the form of beautiful *violet-coloured* fumes, from which it has derived its name. It is very sparingly soluble in water, but much more so in alcohol and æther. It is not inflammable. It forms acids when combined with hydrogen, oxygen, and chlorine, which are respectively named—the *hydriodic*—the *iodic*, and the *chloriodic* acids; and it unites with many of the metals forming *iodurets*. Its acids form salts with the alkalies, earths, and metals; of which, and also of the pharmaceutical preparations derived from these, a very complete catalogue is given in Brera's memoir.

II. *Pharmacology.* We have already stated Dr. Coindet's opinion that the ill effects occasionally found to follow its internal use would be obviated by its external application. Brera, however, informs us that further experience has proved the fallacy of this notion, and assures us that it can be employed internally with equal safety, and with greater effect, except in such cases as require its topical agency. The following are the formulæ most recommended by Professor Brera.

1. *Tincture of Iodine.* Made by dissolving 48 grs. of pure iodine in an ounce of alcohol (at 35.) This is the preparation most frequently used at first by Dr. Coindet, who, as well as Brera, recommends it being used *fresh*, as it is liable to decomposition in a few days. The dose is from five to twenty drops for adults, three times a day. Twenty drops contain about one grain of iodine.

2. *Pills of Iodine*, made by forming one grain of iodine into two pills, with elder-rob and liquorice powder—one to be taken morning and evening.

3. *Iodine Ointment*, made by rubbing up a dram of pure iodine with an ounce of lard, or half a dram of hydriodate of potass with an ounce and a half of lard; the former in the quantity of a scruple, the latter about the size of a filbert, rubbed on the part.

4. *Solution of Hydriodate of Potass*. This preparation is stated to be preferable to any of the foregoing, producing their good effects without their inconveniences. It is formed by dissolving 36 grains of the hydriodate in an ounce of distilled water, and is given in the same dose as the tincture.

5. *Solution of the Ioduretted Hydriodate of Potass*, formed by dissolving 36 grains of the hydriodate and ten grains of pure iodine in ten drams of water. This is said to be a still more efficacious preparation than the preceding, and requires to be given in small doses, viz. five or six drops, three times a day, to begin with.

The following precautions are to be attended to during the administration of iodine :—not to combine it with substances likely to decompose it, and not to give it when the stomach is loaded, but in the morning, a couple of hours before or after dinner, and in the evening. Our author farther recommends the occasional suspension of the medicine, on account of the sometimes sudden supervention of unpleasant effects from it, and to give a dose of magnesia on the day of its suspension, with the view of clearing the primæ viæ. The liquid preparations may be given in any vehicle. Coindet usually employed syrup and water.

### III. *Effects of Iodine on the Living Body.*

A. *On Animals.* We have already observed that both Majendie and Orfila, on the first discovery of iodine, made experiment of its effects on animals. The following are the results derived from the trials of the latter on dogs.

1. Introduced into the stomach in small quantity, it acts as a gentle stimulant, and excites vomiting.

2. In the dose of a dram it invariably destroys the dogs to which it has been administered, (the œsophagus being tied,) producing ulceration of the mucous membrane.

3. In the dose of two or three drams it produces similar effects in the animals whose œsophagus has not been tied, provided they have not vomited for some hours after its ingestion.

4. It is not fatal when applied externally.

5. It seems to act on the human body in the same manner as on animals.

6. It ought to be classed among the corrosive poisons.

B. *On Man.* When iodine is cautiously and gradually introduced into the system, it affects it in a general manner, analogous to that of mercury, but very different in the consequences. The first, and what may be called the *salutary* effects of iodine, are an increase of appetite and of the strength of the pulse; whenever these are produced we must watch with the greatest care that these salutary limits are not exceeded, and the pernicious consequences of an over saturation of the system induced. The complete impregnation of the system is indicated by the change of the above-mentioned increased action of the pulse into decided frequency and quickness—by a sense of heat and irritation of the fauces—pain of the orbits or eye-balls, with obscured vision—pain of the internal ears and gums, (with occasional salivation,) headache, restlessness, loss of sleep, with swelling and pain of the diseased organs, (*e.g.* thyroid and other glands,) and an increase of appetite sometimes to a degree of voracity. In some persons the submaxillary glands become painful and swollen, and a similar state of the mammæ, *with eventual diminution of their natural volume*, takes place in some females. When given from the first in an over-dose, iodine produces a strong burning sensation in the fauces, which frequently extends down the œsophagus to the stomach and whole intestinal canal. In a still higher degree of saturation (or *iodization*, as the author calls it) of the system, to the above-mentioned symptoms succeed very considerable emaciation even in the space of a few days, excruciating pains in the orbits and eyes, with great defect of vision, and similar pains in the diseased parts; the strength vanishes; neuralgic pains are experienced in the stomach, chest, bowels, &c. the sleep entirely fails, and there is obstinate palpitation of the heart, with tremors, convulsions, or palsy of the extremities; to the excessive appetite succeeds complete anorexia, and the factitious disease finally terminates life, in a short time, by universal inflammation of the nervous and vascular systems, (profonde angioitidi e neuritidi.)

Upon the appearance of the milder class of symptoms above-mentioned, the immediate suspension of the medicine (which ought always to be done) sometimes is found sufficient to put a stop to them in a few days. For allaying these deleterious effects, rigorous regimen, copious mucilaginous drinks, and the tepid bath, are recommended; and where the topical affection of the goitre, or other tumors, runs high, fomentations, poultices, leeches, &c. are prescribed; and general bleeding is advised where there exists a high phlogistic state of the whole system.

As these symptoms sometimes show themselves all at once, we ought to be cautious in not too hastily increasing the dose in cases wherein no obvious effects are produced. After the bad symptoms are allayed, the medicine is to be repeated with the same precautions as in the case of mercury and arsenic.

IV. *Medical Effects—or Effects on Disease.* From the preceding history of the effects of iodine on the living system, it must be admitted to be a powerful agent; and the statements formerly made of its efficacy in the cure of bronchocele, prove its potency to be available in the hands of medical men. Of its precise mode of acting on the living system, more especially in the cure of disease, we are hardly well assured. Dr. Coindet says,

"Iodine is a stimulant; it gives tone to the stomach and excites appetite; it neither acts upon the bowels nor kidneys; produces no perspiration, but exercises its action upon the generative system, especially the uterus. If given in a certain dose, and continued for some time, it is one of the most active emenagogues with which I am acquainted; it is, perhaps, from this sympathetic action that it so frequently cures the goitre."—*First Mem. p.* 12. Again—"The experience of two years upon more than two hundred patients, has proved to me, that this remedy is one of the most energetic stimulants we know of the lymphatic system; and the variety of diseases in which I have employed it, (such as goitre, scrophula, enlarged glands of the breast, certain affections of the uterus, some cases of dropsy, &c.) is only apparent, since the whole of these diseases are only lesions of the same system."—*Third Mem. p.* 31.

Professor Brera sums up his opinions respecting the new agent in the following terms:—

"Iodine, then, is, on many accounts, entitled to be classed among the *heroic* remedies, and to obtain a place by the side of mercury. Like mercury, it maintains a permanent action on the system for a considerable time after its administration has been suspended. Powerfully exciting the nervous system, it accelerates the action of the heart and arteries, and restores the functions of the sanguiferous and organic systems when preternaturally affected. It thus produces appetite, fattens the lean, and emaciates the robust.(!) Determining a particular action on the thyroid gland and uterus, it removes the morbid enlargements of the former, promotes scanty, and lessens excessive, menstruation, and even diminishes the size of the mammæ. This last mentioned fact, taken in conjunction with its undoubted efficacy in removing goitre, gives us reason to hope that this medicine may be found beneficial in the cure of organic enlargements of the ovaries and uterus."—*Saggio, p.* 83.

He afterwards adds, that iodine produces its remedial effects without exciting any purgative, diuretic, or diaphoretic action; and that it has no effect on the thyroid gland and uterine functions, when in a *sound* condition.

Many of these sanguine anticipations of the wonders worked, or hereafter to be worked, by iodine, we have no manner of doubt will be disappointed, and are, in truth, little justified by the facts adduced by their very promulgators. Of the great powers of iodine in the cure of bronchocele, we think, we cannot, from the evidence before us, legitimately doubt; and the establishment of this single fact is sufficient, in our minds, to entitle the remedy to the greatest consideration. So rarely, indeed, are we presented, in the practice of medicine, with agents of specific powers, on whose efficacy, in the removal of disease, we can calculate with any thing like certainty,—and so frequently are we left in doubt, even in our most successful cases, whether it was our prescriptions or the spontaneous agency of Nature's restorative powers, that produced the benefit,—that it invigorates, at once, our confidence in medicine, and our zeal in the practice of it, when we fall upon a remedy on which we can pretty confidently rely, even, although the sphere of its operation be confined to a single form of disease. If, then, it be true, that *iodine*, whether in the form of burnt sponge, or kelp, or under a more scientific aspect, is capable of removing a considerable proportion of goitrous tumours, even of many years' standing, we shall be willing to give it a high place in the materia medica, even if its powers should be entirely confined to this class of affections.

In the memoir before us, Professor Brera does not detail his experience of the efficacy of iodine in bronchocele, because, he says, the results obtained by him have been similar to those of Coindet and others. His principal object appears to have been, to try its powers in some other forms of disease, in which it had been proposed or employed by its discoverer, or suggested, to himself, by analogy. The clinical observations detailed by him, are thirteen cases of his own, and four communicated by one of his friends, and consist of one case of (supposed) incipient tabes mesenterica; two cases of vicarious hæmoptysis, and one of vicarious hæmorrhage from the eye; one of chronic dysentery; one, laryngeal phthisis; three of amenorrhœa, and one of dysmenorrhœa; two of swelled submaxillary glands; two of bronchocele; two of scrophulous ophthalmia; and one of scrophulous glandular tumours.

In reviewing the history of these cases, as given by our author, we are compelled to declare, that, in as great a proportion as two-thirds of the whole, there is no proof whatever of the complaints being removed by the iodine; and, that in several, it seems infinitely more probable, that the other means employed at the same time effected the cure. Indeed, we are extremely surprized to find these cases adduced by a practitioner of Professor Brera's experience, as instances of cures effected by the iodine. In the cases of bronchocele, however, and other enlargements of glands, the effect of the remedy appears to have been sufficiently conspicuous; and, in conjunction with Dr. Coindet's experience, fully justify its employment, and with considerable prospect of benefit, in indolent glandular tumours in strumous subjects. We have already devoted too much of our space to permit us to give even an outline of these cases. We must, therefore, refer to the work itself.

In conclusion, we would beg leave to repeat our opinion, that in iodine we have a valuable addition to the materia medica; that its efficacy in the cure of bronchocele, appears to exceed that of every other medicine hitherto employed; that its power in dissipating other chronic glandular tumors seems very probable; and, that it is deserving of a trial in other diseases, more especially in such as bear any analogy to those in which it has been proved to be beneficial.* At the same time, we must recur to the declarations with which we set out, and which can never be too much and too frequently impressed upon the minds of the younger members of the profession, —that it is only by the *study of* DISEASES that professional eminence or usefulness can be obtained,—that a constant hunting after, and great confidence in, medicines of supposed specific powers, is an unfailing indication of a weak understanding,—and, finally, that, according to the expression of the great Boërhaave, *there really is no remedy* "*nisi quod* TEMPESTIVO USU *fiat tale.*"

---

* Since writing the above, Dr. Baron's "Illustration" has come to hand, and in it we find strong reasons for hoping that, in iodine, we possess a medicine of no mean power in resolving tubercles in the lungs, and tubercular affections in other parts of the body. We refer our readers to the analysis of Dr. Baron's work in another part of this number for further particulars.

